# Caffeine Enhances Some Aspects of Physical Performance in Well-Trained Hammer and Discus Throwers

**DOI:** 10.3390/nu16223908

**Published:** 2024-11-15

**Authors:** César Gallo-Salazar, Juan Del Coso, Beatriz Lara, Millán Aguilar-Navarro, Verónica Giráldez-Costas, Francisco Areces, Carlos Revuelta, Jorge Gutiérrez-Hellín, Juan José Salinero

**Affiliations:** 1 Exercise Physiology Laboratory (GIDECS), Faculty of Health Sciences HM Hospitals, Camilo José Cela University, 28692 Villanueva de la Cañada, Spain; blara@ucjc.edu (B.L.); vgiraldez@ucjc.edu (V.G.-C.); fareces@ucjc.edu (F.A.); 2 HM Hospitals Health Research Institute, 28015 Madrid, Spain; 3 Centre for Sport Studies, Rey Juan Carlos University, 28943 Fuenlabrada, Spain; juan.delcoso@urjc.es; 4 Faculty of Health Science, Universidad Francisco de Vitoria, 28223 Pozuelo de Alarcón, Spain; millan.aguilar@ufv.es (M.A.-N.); jorge.gutierrez@ufv.es (J.G.-H.); 5 Faculty of Sport Sciences, Universidad Europea de Madrid, Campus de Villaviciosa de Odón, 28670 Villaviciosa de Odón, Spain; carlos.revuelta@universidadeuropea.es; 6 Sport Training Laboratory (GIRD), Faculty of Sport Sciences, University of Castilla-La Mancha, 45004 Toledo, Spain; juanjose.salinero@uclm.es

**Keywords:** ergogenic aids, athletics, throwing, countermovement jump, side effects

## Abstract

Background: Caffeine is a widely recognized ergogenic aid for enhancing exercise performance. However, its effect on throwing performance has been less studied, yielding contradictory results. Objectives: The main aim of the study was to analyze the potential ergogenic effects of a moderate dose of caffeine (3 mg·kg^−1^ body mass) on vertical jump performance and throwing distance during a simulated competition in trained discus and hammer throwers. Methods: In a randomized, counterbalanced, and repeated measures design, 14 well-trained throwers (9 hammer throwers and 5 discus throwers; age 24.8 ± 6.3 years old, training 14.9 ± 5.0 h per week, competing experience 10.5 ± 6.1 years) performed a countermovement jump (CMJ) test, a modified throw, and a complete throw after the ingestion of 3 mg·kg⁻¹ body mass of caffeine or a placebo. Each participant performed three maximal-effort valid modified throws of his/her respective event (i.e., hammer or discus throw), plus three maximal-effort valid official throws (up to five tries, respectively, in case any attempt was called as foul). Throwing distance was measured according to World Athletics regulations using a metal tape, while release speed was assessed with a radar device. After the performance measurements, participants completed a form about side effects prevalence. Results: Caffeine, compared to placebo, increased throw distance (3.0 ± 5.1%, *p* = 0.048) and speed release (5.7 ± 8.7%, *p* = 0.03) for the complete throw, and distance (3.6 ± 4.4%, *p* = 0.01) and speed release (4.8 ± 7.4 %, *p* = 0.01) for the modified throw. Caffeine ingestion did not significantly improve jump height (1.1 ± 4.3%, *p* = 0.28), although it improved force and power on braking and the propulsive phases of the CMJ (*p* < 0.05). Caffeine only increased the prevalence of activeness (*p* < 0.05). Conclusions: An acute moderate dose of caffeine enhanced hammer and discus throw performance in well-trained throwers during a simulated competitive setting, with minimal adverse side effects.

## 1. Introduction

Athletes commonly use sports supplements to enhance performance, promote training adaptations, and support recovery [1]. However, only a limited number of sports supplements have been scientifically validated as effective for enhancing athletic performance. Evidence-based supplements such as caffeine, bicarbonate, beta-alanine, nitrate, and creatine may provide performance benefits that are influenced by the specific characteristics of the competition, the athlete’s fitness level, dosage, and individual tolerance [2,3,4]. While nitrate, beta-alanine, and bicarbonate may be beneficial for longer, aerobic-based events, caffeine, due to its multifaceted mechanisms of action, can provide advantages across a range of exercise durations, from short to long [2]. These benefits are driven by mechanisms such as adenosine receptor antagonism, elevated endorphin release, enhanced neuromuscular function, increased vigilance and alertness, and a decreased perception of effort during exercise [4]. Caffeine is a widely used ergogenic aid for enhancing exercise performance due to its accessibility and the general awareness among athletes of the benefits of acute caffeine supplementation. Most athletes used this substance in competition (≈3 out of 4 according to urine samples obtained from anti-doping controls [5]). This wide use is based on solid evidence of its ergogenic properties on a wide variety of sports and exercise activities [6,7,8]. While caffeine effects on aerobic-based exercise were clearly stated decades ago, research about strength-related exercise was scarce until recently [9]. However, there is also recent solid evidence about the ergogenic properties of caffeine on strength and muscular power [7,10]. Previous systematic reviews have demonstrated that a moderate dose (≈3–6 mg·kg^−1^) of caffeine can improve isokinetic muscular strength (particularly manifested in knee extension and at higher angular velocities [11]) and the rate of force development [10], maximal strength, muscular endurance, velocity, and power in different resistance exercises [12]. Nevertheless, most studies about this topic have been developed in laboratory/machine strength tests, so, ecological studies in the sport context are still scarce. Throws in athletics (e.g., hammer and discus throw), compared to other sports, require a unique combination of explosive power, precise technique, and timing, making them more complex than other sports. Thus, while in other endurance disciplines, caffeine can exert its effects mainly on the central nervous system via the antagonism of adenosine receptors, in power sports, the improved muscle contraction could be responsible for enhanced exercise performance [7,13].

Caffeine effects on throwing performance have received less attention, and contradictory results have emerged in previous research [14,15,16]. In addition, most research has tested the effect of caffeine on medicine ball throws [14,17] and bench press throws [15,16] that do not represent real sporting events. For example, Rocha et al. [14] did not find ergogenic effects on medicine ball throws (2 kg, from the chest) in handball players with a dose of 5 mg·kg^−1^ of caffeine. On the contrary, Sabol et al. [17] found an ergogenic effect in a 9 kg medicine ball throw with a dose of 6 mg·kg^−1^ of caffeine in recreationally active male participants. In this latter study by Sabol et al., 2 and 4 mg·kg^−1^ of caffeine were ineffective in improving throwing distance, suggesting the need of ingesting higher doses to obtain the benefits of caffeine in medicine ball throws. Additionally, caffeine increased mean velocity and mean power output during a bench press throw test with 30% [15] and 70% of 1RM [16]. Last, a systematic review analyzing the effect of caffeine on ballistic exercises (i.e., throwing activities [17]) revealed that caffeine ingestion enhanced throwing performance, but this investigation compiled data of different tests with very diverse characteristics (medicine ball throw, backward throw, shot put, bench press throw, handball throw, and ballistic push up) and athletes of very different conditions (shot putters, handball players, and resistance-trained participants). In sports, only a few disciplines focus on achieving maximum distance in a throwing event. Among these, the most well-known are the throwing events in athletics, which include shot put, discus, hammer, and javelin. Each of these events requires athletes to throw an implement with distinct weights and characteristics within a confined throwing area. The nature of these athletic throws is significantly different from other types of throws, such as those in handball and medicine ball exercises. Therefore, based on the above-mentioned information, it is unclear whether athletes in throwing sports, such as throwing events in athletics, could benefit from caffeine supplementation.

Throwing performance in athletics is primarily based on dynamic strength, speed, and power, applied over a full range of multiple body joint movements [18]. This performance is also limited by the specific sequence of the thrower’s movements in various phases of the throw, up until release, and in the recovery that follows to prevent a foul throw [19,20,21]. In a hammer throw, the athlete swings a heavy ball (7.6 kg for men and 4 kg for women) attached to a wire and handle (i.e., the hammer) in circular motions to gain momentum. The most common hammer throw technique consists of building speed with up to four spins on a 2.13-m-diameter circle before releasing the hammer. The hammer thrower uses the action of the leg muscles to gain momentum, which is then transferred to the hammer through the trunk and arm muscles, thus obtaining an increase of the linear release velocity [19]. In a discus throw, the athlete spins within a circular area (2.5 m of diameter) to build momentum before releasing a heavy disc into the air (2 kg for men and 1 kg for women). In both types of throws, release velocity is the most critical factor for maximizing distance. The throwing motions are designed to increase this velocity through coordinated actions of the lower limbs, trunk, and upper limbs [19,21]. So, far from the isolated effect of muscular strength and power in throwing a medicine ball from the chest in a sitting position or throwing the bar in a bench press test, throws in athletics are very complex tasks, and performance in these events requires a well-developed movement technique [22]. Additionally, throwers need to generate large amounts of force on the throwing implements in short time windows, usually between 150 and 240 milliseconds, generating high rates of force development [22]. To the authors’ knowledge, only two previous studies have analyzed the ergogenic effect of caffeine in athletics throws, both in shot put [23,24]. First, Bellar et al. [23] found an ergogenic effect of a low dose of caffeine (i.e., 100 mg, not individualized by body mass) in trained college shot putters. They did not use an ecological shot put, but a standing shot put (i.e., a less complex task). These authors found that caffeine produced better shot put performance throughout six throws. After this, Giraldez-Costas et al. [24] found an ergogenic effect of 3 mg·kg^−1^ of caffeine in several aspects of shot put performance in trained shot putters (i.e., standing shot put, countermovement jump), but no significant effect was found in the complete shot put. These conflicting results, along with the limited research on other throwing disciplines in athletics, led to the purpose of this study: to analyze the potential ergogenic effects of a moderate dose of caffeine (3 mg·kg⁻¹ body mass) on vertical jump performance and throwing distance during a simulated competition in trained discus and hammer throwers. Based on the literature, we hypothesized that caffeine, compared to placebo, would improve variables of vertical jump as well as throwers’ performance in a simulated competition (i.e., higher maximum throwing distance) while some side effects from the ingestion of this stimulant would likely emerge.

## 2. Materials and Methods

### 2.1. Participants

Fourteen well-trained throwers (five female and four male hammer throwers, and three female and two male discus throwers; age 24.8 ± 6.3 years, body mass 94.5 ± 16.8 kg, height 177.4 ± 11.7 cm, training volume 14.9 ± 5.0 h per week, competing experience 10.5 ± 6.1 years) volunteered to take part in the study. All of them were ranked among the top 12 in their respective categories at the national level. They all were requested to fulfil a questionnaire about their habitual caffeine ingestion [25], whose results showed that they were low or mild caffeine consumers (1.4 ± 0.7 mg·kg^−1^ per day; all <3 mg·kg^−1^ per day [26]).

All the participants met all the following inclusion criteria: (a) to be 18+ years of age; (b) to have been free of cardiovascular, pulmonary, and musculoskeletal injuries six months before the study; and (c) to have at least five years of high-level competition experience. All the participants were fully informed about the protocol and signed a written consent before the study. The study was conducted according to the guidelines of the Declaration of Helsinki and approved by the Camilo Jose Cela University’s Ethics Committee (ref. 28.1.2021CEI-UCJC; Comité de Ética de la investigación de la Universidad Camilo José Cela, 2 February 2021).

### 2.2. Experimental Design

Participants took part in a two-day, double-blind, randomized, counterbalanced, placebo-controlled experimental crossover study to evaluate their lower limb neuromuscular performance (through countermovement jumps, or CMJs) and their throwing performance (through complete and modified throws). The experiment had a seven-day gap between trials to guarantee substances’ full washout, participants’ total recovery, and replicate training microcycle. Forty-five min prior to the start of each experimental trial, participants ingested either 3 mg·kg^−1^ of body mass of caffeine (100% purity; Harrison Sport Nutrition [HSN] Store, Granada, Spain) or the same amount of placebo (cellulose; 100% purity, Guinama, Spain). Identical opaque capsules and 200 mL of water were used for substance intake, which occurred 45 min prior to the start of measurements in front of the researchers to ensure proper absorption. Participants were asked to refrain from strenuous exercise and stimulant drink intake, to replicate dietary/drink habits 24 h before each experimental trial, and to avoid caffeine ingestion (including coffee, tea, chocolate, and any supplements containing caffeine) 48 h before the experimental procedures. Compliance with these recommendations was verbally confirmed in each experimental session. Weather conditions were alike (temperature 8.2–10.4 °C; relative humidity 40.4–45.1%), and weekday and time of day (i.e., afternoon) were also the same in both trials to guarantee test reproducibility.

### 2.3. Intervention

One week before the onset of the study, participants took part in a familiarization session wherein all the tests were replicated following the same instructions, procedures, and time order as in subsequent trials.

Every trial day, participants arrived at the testing facility 60 min before the beginning of the tests (between 12:00 and 16:00 p.m.). Then every participant ingested their capsule and underwent a 30 min standardized competition warm-up following their own routine (e.g., upper and lower body mobility, strength exercises, and specific throws). Forty-five minutes after the ingestion, throwers started to perform the following tests.

#### 2.3.1. Countermovement Jump Test

CMJs were performed using a wireless dual force plate system with a sample rate of 1000 Hz (Hawkin Dynamics Inc., Westbrook, ME, USA). The data were automatically low-pass filtered with a 50 Hz cut-off. Force plates were connected via Bluetooth to an Android tablet (Android, Palo Alto, CA, USA), where the Hawkin Dynamics Inc. software (HD app, version 8.6.0) automatically transferred data via Wi-Fi to the HD cloud server for their subsequent download and analysis.

After a brief specific jump warm-up (e.g., five submaximal CMJs), each participant was instructed to stand upright over the force plates with their hands placed on their hips. Then, according to procedures followed in previous research [22], they were encouraged to jump “as high and as fast as possible” three times, interspersed by 1 min of rest between jumps. Different metrics involved in both the eccentric (e.g., braking) and concentric (e.g., propulsive) phases of CMJs were recorded -peak relative force (%), peak relative power (W·kg^−1^), phase time (s), and average velocity (m·s^−1^), as well as jump height (cm) and relative force at min displacement (N·kg^−1^). The best values from each variable were recorded for subsequent analysis.

#### 2.3.2. Throwing Tests

After the CMJs, participants went to an outdoor official throwing facility and took part in a simulated competition, where they were encouraged to perform three maximal-effort valid modified throws, plus three maximal-effort valid official throws (up to five tries, respectively, in case any attempt was called as foul). Official throws for both discus and hammer events were performed according to IAAF/World Athletics rules. Hammer weights were 7.26 kg and 4 kg for men and women, respectively; and discus weights were 2 kg for men and 1 kg for women. The modified throws were different from the official throws for each throwing event (i.e., discus or hammer throwing). The hammer-modified throw was called heavy throw since it consisted of performing the same official throw with an increased weight of the hammer (from 7.26 kg to 8 kg for males, and from 4 kg to 5 kg for females). On the other hand, the discus modified throw was called standing throw (i.e., throwers were neither allowed to do the flight phase nor spin around the circle during the throw). The discus throwers then started directly from the delivery phase, or power position [21,27], where the throwers had both feet on the ground and could do a full trunk rotation before the delivery. These heavier implement throws (hammer) and throws from place (discus) are commonly used as assistive exercises and have positive effects on training transfer to competition [28]. Official discus weights (i.e., 2 kg for males and 1 kg for females) were used for modified throws. Resting time between attempts ranged from three to five minutes, while the remaining competitors were throwing. Before and after the modified throw, a specific warm-up throwing routine was carried out by the participants to ensure optimal performance. The total throw distance (m) and throwing speed release (km·h^−1^) of every throw were measured, while only the maximal values (i.e., from both modified and complete throws, respectively) were subsequently used for the analysis. Two highly qualified coaches supervised the throwing session and took part in the data collection. Artefact velocity was measured by a radar gun (ATS, Stalker, Salt Lake City, UT, USA), placed 3 m behind the thrower during the attempts.

#### 2.3.3. Side Effects

The morning after conducting the tests, participants received a link to complete an electronic form regarding potential side effects (Google Forms). In this survey, they had to indicate on a dichotomous scale (i.e., yes/no) the presence or absence of each possible side effect, such as nervousness, digestive problems, and difficulty sleeping. This questionnaire had previously been used to assess the side effects of caffeine ingestion in the sports domain [29].

### 2.4. Statistical Analysis

An a priori sample size estimation for a paired sample t-test was made based on previous studies in the field [23,24]. The effect size (ES) obtained with caffeine on standing shot put in Bellar et al.’s investigation [23] showed that at least 11 participants were needed to investigate the potential ergogenic effect of caffeine with an ES of 0.996. Conversely, the ES obtained on the standing shot put throw in Giraldez-Costas et al.’s investigation [24] showed that at least 13 participants were needed with an ES of 0.87. Both estimations were tested with a two-tailed paired sample t-test (1 − β = 0.8; α = 0.05) using Jamovi software (version 2.3.28) [30].

Data are presented as mean ± standard deviation (SD), mean differences (95% IC), and % of change caffeine vs. placebo. Statistical analyses were performed using Jamovi software (version 2.3.28). The Shapiro–Wilk test was used to confirm the normality of the raw data, where all variables presented normal distribution. A two-way analysis of variance (ANOVA) for independent measures was conducted to examine the main effects of sex (male vs. female), event (hammer vs. discus), and their interaction (sex × event) on each dependent variable. There was no effect from sex, throwing event, or the interaction of both on any variable (caffeine vs. placebo) increment (*p* > 0.05), so the entire sample was analyzed as a single group. A t-test for paired samples was performed to compare between trials (caffeine vs. placebo). Additionally, Cohen’s *d* effect size (ES) was also used for a better interpretation of the results. The effect sizes were considered as trivial (<0.2), small (<0.6), moderate (<1.2), large (<2), or very large (>2) [31]. For the side effects analyses, changes in % of affirmative responses between trials were analyzed through the McNemar test. The statistical significance was set at *p* ≤ 0.05.

## 3. Results

Table 1 shows the effect of 3 mg·kg^−1^ of body mass of caffeine, or placebo, on countermovement jump performance. Compared to placebo, caffeine improved diverse variables from the countermovement jump. Concretely, variables from the eccentric phase, such as peak relative braking force (4.1 ± 4.9%, *p* = 0.01, moderate ES = 0.80) and time spent on braking phase (−5.0 ± 9.2%, *p* = 0.04, moderate ES = 0.61)]; from the concentric phase, such as peak relative propulsive force (2.8 ± 3.9%, *p* = 0.02, moderate ES = 0.68), peak relative propulsive power (1.7 ± 3.0%, *p* = 0.03, moderate ES = 0.63), and average propulsive velocity (1.7 ± 2.7%, *p* = 0.04, moderate ES = 0.60); as well as from in-between phases, such as relative force at minimum displacement (4.0 ± 4.9%, *p* = 0.01, moderate ES = 0.80).

Nonetheless, caffeine did not significantly improve total jump height (1.1 ± 4.3%, *p* = 0.28, small ES = 0.30), average braking velocity (2.5 ± 9.0%, *p* = 0.30, small ES = 0.29), peak relative braking power (5.1 ± 14.1%, *p* = 0.14, small ES = 0.41), or time spent on the propulsive phase (−2.1 ± 7.2%, *p* = 0.33, moderate ES = 0.60), although most of the participants on these three variables (i.e., 8, 9, 10 and 7 participants out of 14, respectively) individually received an increment with caffeine, compared to placebo.

**Figure 1:**
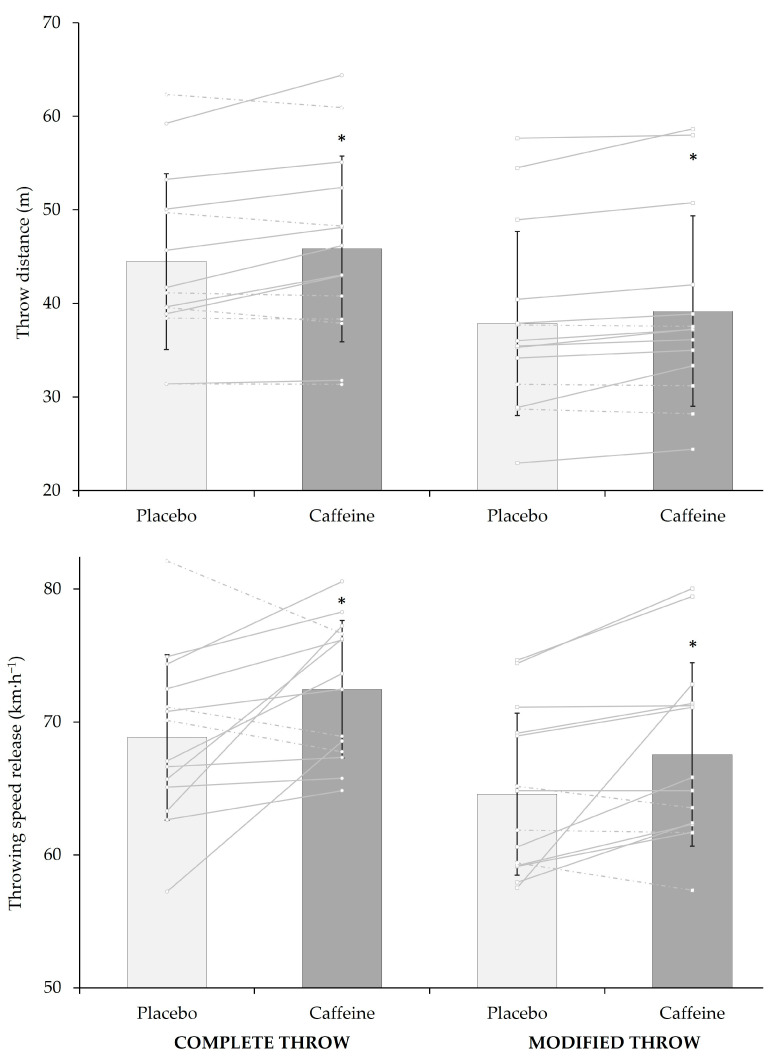
Individual responses for throwing distance (m) and speed release (km·h^−1^) in a complete throw (left panel) and a modified throw (right panel) after the ingestion of 3 mg·kg^−1^ of body mass of caffeine or placebo. Columns represent means, and black vertical lines represent standard deviations. Grey lines represent individual changes (straight lines depict increments in performance with caffeine ingestion, with respect to placebo, whilst dashed lines depict decrements in performance with caffeine). * Significantly different from placebo at *p* < 0.05.

The effect of caffeine on both the distances obtained and speed release in the different throwing events can be observed in Figure 1. Caffeine, compared to placebo, increased throwing distance (3.0 ± 5.1%, *p* = 0.048, small ES = 0.58, Figure 1) and speed release (5.7 ± 8.7%, *p* = 0.03, moderate ES = 0.65, Figure 1) for the complete throw. Similarly, the same findings were obtained for the modified throw, where caffeine, compared to placebo, significantly increased throw distance (3.6 ± 4.4%, *p* = 0.01, moderate ES = 0.90, Figure 1) and speed release (4.8 ± 7.4%, *p* = 0.01, moderate ES = 0.80, Figure 1).

Table 2 shows the prevalence of side effects in the hours following the ingestion of substances in both trials. Only activeness had a significantly higher prevalence with caffeine ingestion (*p* < 0.05), compared to placebo.

## 4. Discussion

The main findings of the study showed that, compared to a placebo, caffeine improved total throw distance and speed release in both modified and complete throws (Figure 1). Moreover, compared to placebo, caffeine increased meaningful variables related to the braking (i.e., peak relative force and time) and propulsive (i.e., peak relative force, peak relative power, and average velocity) phases of a CMJ, but did not improve jump height performance (Table 1). Caffeine ingestion only showed a higher prevalence of activeness (Table 2).

To the best of the authors’ knowledge, this is the first study to specifically investigate the ergogenic effects of caffeine on performance in hammer and discus throwers. The research about the effects of caffeine on throwing performance has mainly employed simple motor tasks, such as medicine ball throws [17] and bench press throws [16]. Only two previous studies have analyzed caffeine’s effects on throwing events of athletics, both on shot put [23,24]. Both Bellar et al. [23] and Giraldez-Costas et al. [24] found improvements of 6.0% (*p* = 0.05; moderate ES = 1.00) and 2.6% (*p* = 0.01; moderate ES = 0.87), respectively, on the standing shot put performance. However, only Giraldez-Costas et al. studied the effect of caffeine over the complete shot put, finding a non-statistically significant improvement of 1.0% compared to placebo (small ES = 0.33; *p* = 0.26). However, the technique for shot put differs significantly from that of discus and hammer throws. Hammer and discus throws involve complex movements, including rotation and gliding, to maximize performance through dynamic displacement, while the standing shot put relies on a simpler standing technique that emphasizes explosive power from a stationary position. In the current study, caffeine significantly improved the throwing speed release by 5.7% and 4.8% in both complete and modified throws, resulting in a 3.0% and 3.6% of further distance reached, respectively. Individually, 57.1% and 78.6% of the participants improved their total distance after caffeine ingestion in both the complete and modified throws, respectively. The results of this study are novel, showing that high-performance throwers may benefit from caffeine supplementation to enhance their performance in complex disciplines like hammer and discus throws. Additionally, the outcomes align with previous research demonstrating the ergogenic effects of caffeine on physical performance, particularly in explosive and high-intensity activities [10,32], and also on throwing performance [23,24,32]. Collectively, in hammer and discus throwing, caffeine may act as an ergogenic aid, enhancing performance in both training and competitive settings.

The effects of caffeine on jumping ability have been extensively studied in the scientific literature [33,34,35]. Multiple studies have analyzed whether caffeine can enhance jumping capacity, particularly in the countermovement jump (CMJ), as a simple and standardized measure of evaluating an athlete’s neuromuscular performance. Improvements of around 2 to 5% have been observed in different trained athletes [34,36,37,38,39]. However, other studies have found smaller and even non-significant improvements on jump performance with caffeine [40]. Nevertheless, the general trend indicates that caffeine enhances performance in the CMJ [35]. In our case, 8 out of 14 throwers improved their jump height, 6 of which ranged from 1.5 to 8.5%, and 3 worsened jump performance with caffeine (ranging from 0.4 to 0.7%). Notwithstanding this, differences in jump height between caffeine and placebo did not reach statistical significance. While some studies have reported that high doses are necessary to improve jumping performance (i.e., 6 mg·kg^−1^ and not 3 mg·kg^−1^) [41], moderate doses (i.e., 3 mg·kg^−1^) have demonstrated ergogenic effects on jump performance in several studies [24,39,42]. Indeed, Matsumura et al. [34] compared 1, 3, and 6 mg·kg^−1^ of caffeine and found that both low and moderate doses enhanced CMJ performance, without significant differences between doses. Concretely in throwers, contrary to our findings, Giraldez et al. [24] found that a moderate dose of caffeine (e.g., 3 mg·kg^−1^), increased the jump height 5.0% compared to a placebo. Additionally, this enhanced jump performance with caffeine was accompanied by an increase of 6.0% in the standing throw distance for shot putters. In our study, even though jump height was not enhanced with caffeine, both throwing speed release and total throwing distance improved for complete and modified throws were superior with caffeine. Thus, these outcomes may suggest that throwers may obtain benefits from caffeine in their disciplines even when this substance does not improve simpler tasks such as CMJ performance.

Nevertheless, caffeine, as a psychoactive substance, can be associated with adverse side effects that should be considered before deciding to use it as an ergogenic aid in sports. In our study, we only found an increase in the prevalence of activeness (71.4% with caffeine vs. 0% with placebo; *p* < 0.01). Even though nervousness did not present a statistically significant effect, it showed a remarkable increase in its prevalence (50% with caffeine vs. 7.1% with placebo, *p* = 0.07). These findings are consistent with previous studies using similar doses of caffeine [29], suggesting that these moderate doses could be considered safe, although an individual case-by-case approach would be advisable before supplementing with caffeine [4,7]. Studies comparing higher doses have found that the prevalence of these side effects increases considerably with larger doses [43], so it would be reasonable to recommend the use of moderate doses when deciding to use caffeine as an ergogenic aid. However, any sports supplement, including caffeine supplementation, should be thoroughly trialed in a training context, a simulated competition, or a low-level real competition to assess individual responses to the substances [2]. Testing the potential benefits of caffeine in simulated competitions not only assesses its effectiveness in enhancing athletic performance, but also evaluates any adverse side effects that may arise from caffeine supplementation. This is crucial for this psychoactive substance, as its effects can be beneficial for some athletes while proving counterproductive for others. For instance, in our study, caffeine ingestion led to increased activeness (Table 2). While this heightened activation can be advantageous for some athletes preparing for maximal efforts, it may not be as beneficial for others, particularly in complex technical disciplines like throwing events in athletics. Qualitative analysis of the technique could be incorporated in future studies to identify the potential relation between this increased activeness or nervousness in technical alterations and failed throws. By evaluating caffeine’s ergogenic effects in simulated trials, coaches and athletes can make informed decisions about its use in specific competitions.

This experimental study presents some limitations that deserve discussion. First, plasma and urine caffeine levels were not measured; thus, compliance with the recommendation to abstain from caffeine or caffeine-containing substances throughout the experimental conditions could not be verified. Secondly, these results were found in highly trained athletes. So, the results may differ at lower performance levels, as the technical movement may not be as well-automated. Finally, the heterogeneity of the sample (i.e., it consisted of different genders [six male and eight female] and disciplines [e.g., nine hammer and five discus throwers]) athletes could lead to a possible bias in the results. To avoid this potential distortion, a two-way analysis of variance was conducted, and it confirmed that the sample could be used as a single group, since no group (e.g., gender, discipline, or their interaction) effects had been observed for any of the studied variables. Still, further investigations are needed to confirm that acute caffeine ingestion produces a similar ergogenic effect in male and female throwers.

## 5. Conclusions

The main outcomes of the present study indicate that the intake of a moderate dose (e.g., 3 mg·kg^−1^ of body mass) of caffeine increased hammer and discus throwers’ performance in a simulated competitive setting, since it improved speed release and overall throwing distance, with minimal adverse side effects. These findings provide useful information for athletes and coaches in search of alternative ways to improve their results. In this regard, acute caffeine supplementation may be viewed as an ergogenic aid for high-performance throwers; however, the risk-to-benefit ratio should be evaluated on an individual basis.

## Figures and Tables

**Table 1:** Countermovement jump metrics after the ingestion of 3 mg·kg^−1^ of body mass of caffeine or placebo.

	Placebo	Caffeine	Mean Differences (95% CI)	ES
Jump height (cm)	31.2 ± 7.9	31.6 ± 8.0	0.40 (−0.40, 1.20)	0.30
Peak relative braking force (%)	249.6 ± 23.6	260.3 ± 21.8 *	10.70 (306 ± 18.34)	0.80
Peak relative braking power (W·kg^−1^)	−18.12 ± 4.9	−19.34 ± 5.3	−1.22 (−2.91 ± 0.47)	0.41
Average braking velocity (m·s^−1^)	−0.79 ± 0.13	−0.81 ± 0.11	−0.02 (−0.07 ± 0.02)	0.29
Braking phase (s)	0.14 ± 0.02	0.13 ± 0.02 *	−0.007 (−0.01 ± 0.00)	0.61
Relative force at min displacement (N·kg^−1^)	249.8 ± 23.5	260.4 ± 21.6 *	10.61 (2.97 ± 18.25)	0.80
Peak relative propulsive force (%)	256.0 ± 21.6	263.5 ± 22.1 *	7.48 (1.16 ± 13.79)	0.68
Peak relative propulsive power (W·kg^−1^)	48.19 ± 7.84	49.12 ± 8.27 *	0.93 (0.08 ± 1.79)	0.63
Average propulsive velocity (m·s^−1^)	1.52 ± 0.14	1.55 ± 0.15 *	0.03 (0.00 ± 0.05)	0.60
Propulsive phase (s)	0.24 ± 0.03	0.23 ± 0.03	−0.004 (−0.01 ± 0.00)	0.60

Data presented as mean ± SD, and 95% CI of the mean difference between treatments. CI = confidence interval; ES = effect size; min = minimal. * Significantly different from placebo at *p* < 0.05.

**Table 2:** Prevalence of side effects in the following hours to the ingestion of 3 mg·kg^−1^ of body mass of caffeine or placebo.

	Placebo (%)	Caffeine (%)	*p*
Nervousness	7.1	50.0	0.07
Activeness	0.0	71.4 *	<0.01
Irritability	7.1	7.1	1.00
Muscle damage/Stiffness	7.1	14.3	1.00
Headache	7.1	7.1	1.00
Gastrointestinal problems	7.1	7.1	1.00
Increased urine production	7.1	21.4	0.63
Sleep difficulties	0.0	21.4	0.25

Data presented as % of affirmative responses. * Statistically significant effect versus placebo at *p* < 0.05.

## Data Availability

The data are not publicly available due to privacy reasons, since they are part of an ongoing project. However, the data will be shared upon request to the author for scientific purposes or for systematic reviews and meta-analyses (C.G.-S.).
